# Large mammal telomere length variation across ecoregions

**DOI:** 10.1186/s12862-022-02050-5

**Published:** 2022-08-29

**Authors:** Christian Fohringer, Franz Hoelzl, Andrew M. Allen, Claire Cayol, Göran Ericsson, Göran Spong, Steven Smith, Navinder J. Singh

**Affiliations:** 1 Department of Wildlife, Fish and Environmental Studies, Swedish University of Agricultural Sciences, 90183 Umeå, Sweden; 2 Konrad Lorenz Institute of Ethology, University of Veterinary Medicine, Savoyenstraße 1, 1160 Vienna, Austria; 3 Department of Animal Ecology, Netherlands Institute of Ecology (NIOO-KNAW), Droevendaalsesteeg 10, 6708PB Wageningen, The Netherlands; 4 Department of Animal Ecology and Physiology, Radboud University, 6500GL Nijmegen, The Netherlands

**Keywords:** *Alces alces*, Biomarker, Chronic stress, Human modification, Life history, Telomere associations

## Abstract

**Background:**

Telomere length provides a physiological proxy for accumulated stress in animals. While there is a growing consensus over how telomere dynamics and their patterns are linked to life history variation and individual experience, knowledge on the impact of exposure to different stressors at a large spatial scale on telomere length is still lacking. How exposure to different stressors at a regional scale interacts with individual differences in life history is also poorly understood. To better understand large-scale regional influences, we investigated telomere length variation in moose (*Alces alces*) distributed across three ecoregions. We analyzed 153 samples of 106 moose representing moose of both sexes and range of ages to measure relative telomere lengths (RTL) in white blood cells.

**Results:**

We found that average RTL was significantly shorter in a northern (montane) and southern (sarmatic) ecoregion where moose experience chronic stress related to severe summer and winter temperatures as well as high anthropogenic land-use compared to the boreal region. Our study suggests that animals in the northern boreal forests, with relatively homogenous land use, are less disturbed by environmental and anthropogenic stressors. In contrast, animals in areas experiencing a higher rate of anthropogenic and environmental change experience increased stress.

**Conclusion:**

Although animals can often adapt to predictable stressors, our data suggest that some environmental conditions, even though predictable and ubiquitous, can generate population level differences of long-term stress. By measuring RTL in moose for the first time, we provide valuable insights towards our current understanding of telomere biology in free-ranging wildlife in human-modified ecosystems.

**Supplementary Information:**

The online version contains supplementary material available at 10.1186/s12862-022-02050-5.

## Background

Human-induced rapid environmental change is creating novel stressors for animals and their populations [1]. These external changes cascade via physiological mechanisms affecting long-term survival and fitness in wild animals. In particular, exposure to anthropogenic perturbations (resource extraction, infrastructural developments, hunting, and pollution) combined with environmental stressors (competition over resources, disease, or thermal stress) may activate the hypothalamic–pituitary–adrenal (HPA) axis of animals resulting in increased stress hormone levels [2–4]. Continued activation of the HPA axis beyond baseline levels can affect the metabolic system of the organism via increased oxidative damage from reactive oxygen species (ROS), and induce a state of chronic stress [5]. The (TTAGGG)n repeats that constitute vertebrate telomeres are particularly vulnerable to oxidative attack [6]. Telomeres, i.e., the non-coding ends of linear chromosomes, are considered to play a fundamental role in the protection of the structural integrity of chromosomal DNA and in the regulation of cellular senescence [7, 8]. Thus, they have the potential to serve as a molecular biomarker to determine individual physiological state and past environmental experiences [9, 10]. Shorter telomeres and elevated shortening rates are typically associated with stress and senescence [7, 9, 11, 12]. Angelier et al. [13] reviewed studies determining the relationships between how different stressors can influence telomere associations in wild vertebrates. Specifically, environmental factors such as water temperature [14], weather [15], habitat quality [16–18] as well as infectious diseases [19] were linked to altered telomere length in wildlife.

Ecoregions provide an ideal spatial scale to examine differences in metabolic expenditure and chronic stress expression as they offer a global categorization representing distinct units of biological diversity and its association with climatic conditions [20]. Distinct ecoregions also encompass differences in anthropogenic pressures, food availability and weather. Differences in the degree of exposure to different environmental conditions (including an array of stressors) can potentially cause chronic stress in organisms occupying ecoregions where they experience repeated triggering of the HPA axis beyond full recovery during the annual and seasonal cycles. Yet, comparative studies of chronic stress responses, or its indicators, across biogeographic regions are largely absent. This is especially true as data on multiple individuals and populations distributed across large spatial scales are not often compared.

The main objective of this study is to compare relative telomere length (RTL) across ecoregions and therefore identify how levels of anthropogenic and environmental stress may correlate with RTL of individuals across multiple populations. Our focal study species is the moose (*Alces alces*) across the main three ecoregions in Sweden. Shorter term stress in response to anthropogenic and environmental stressors have been demonstrated in moose previously [21] and their longevity (up to ~ 20 years) makes them an ideal model species to also evaluate accumulated stress across an individual’s life span and to compare these across ecoregions. In addition, moose are a cold-adapted species and are susceptible to heat-stress at ambient temperatures above 14–17 °C [22, 23] during summer and above − 5–0 °C during winter [22], meaning they may be particularly susceptible to temperature changes brought about by climate change. In combination with a known higher parasite burden [24, 25], higher hunting pressure and higher inter-species competition [26, 27] that moose are exposed to in their southern range, we expect the chronic stress burden of moose to decrease with increasing latitude. However, moose at high altitudes, i.e., montane tundra habitat, may experience stress due to other factors, such as high snow depth [28]. With this study we analyze RTL in moose for the first time and examine how it reflects chronic stress of individuals experiencing varying levels of environmental factors and anthropogenic impacts across large spatial scales.

## Results

### Geographic variation in climate and land use

**Fig. 1:**
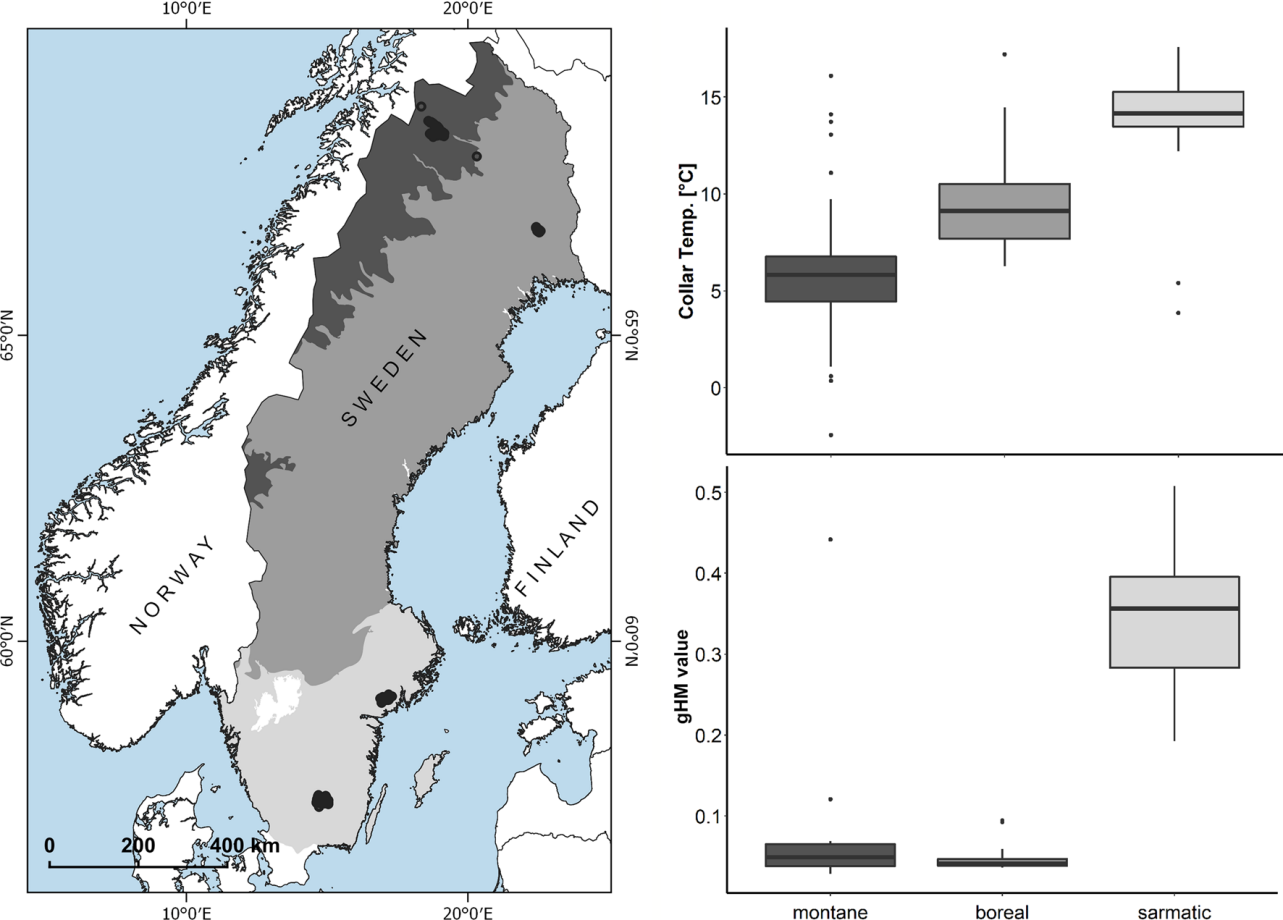
**A** Capture locations of 106 moose in three ecoregions (dark grey = montane birch forest and grasslands, grey = boreal forest, light grey = sarmatic mixed forest). The map was created in Quantum GIS, version 6.10.6 (QGIS.org, 2020). Right: Mean annual GPS-collar temperature **B** and corresponding mean global human modification (gHM) values extracted based on the annual GPS track **C** of all individuals distinguished by ecoregion

In line with our general hypothesis, we observed a marked difference in GPS-collar recorded temperature [F(2,149) = 111.4, *P*-value < 0.001] and land-use intensity [F(2,150) = 404.4, *P*-value < 0.001] that moose experienced in each ecoregion based on their annual movements (Fig. 1). Mean annual temperature (based on GPS-collar temperature: T_c_) was 5.44 ± 4.08 °C in the montane, 9.80 ± 2.71 °C in the boreal and 14.16 ± 2.10 °C in the sarmatic ecoregion (Fig. 1). Land use intensity follows a similar trend with low mean global Human Modification (gHM, [29]) values encountered by moose in the montane (0.06 ± 0.05) and boreal (0.05 ± 0.02) but high mean values (0.34 ± 0.08) encountered in the sarmatic ecoregion (Fig. 1).

### Relative telomere length

**Fig. 2:**
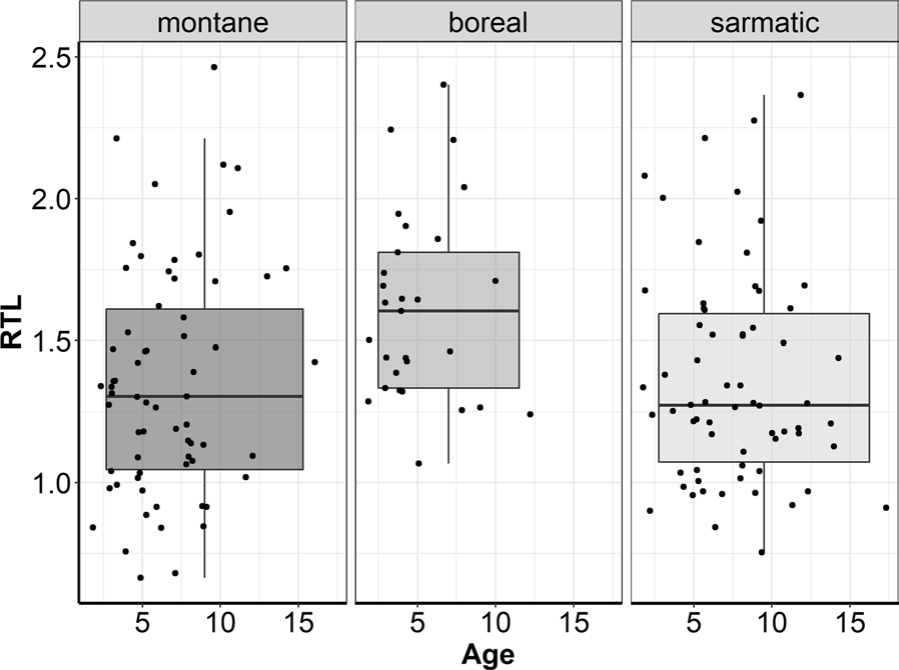
Average observed relative telomere length (RTL) of 153 samples in three ecoregions (n_montane_ = 58, n_boreal_ = 29, n_sarmatic_ = 66) from 106 animals. Animal age was included for each sample as black dots, despite having been excluded from the final model

The ecoregions variable explained significant differences in RTL as per the final model (Fig. 2; Table 1). Compared to the boreal region, RTLs were significantly shorter in the sarmatic study areas (1.42 [1.31, 1.53] 95% CI) in southern Sweden. Additionally, shorter RTLs were also observed in the northern montane area (1.35 [1.20, 1.44] 95% CI) compared to the boreal region (1.63 [1.49, 1.76] 95% CI). Sample storage time was negatively correlated with RTL. Based on linear mixed effect model selection, sex and age of animals did not influence RTL significantly and were subsequently removed as explanatory variables (Additional file 1: Table S1, Fig. S1–2). Pregnancy and the number of calves at heel did not affect RTL (Additional file 1: Fig. S3).

**Table 1:** The best linear mixed effect model showing the relationship between relative telomere length of moose individuals (N = 106), the three considered ecoregions in Sweden, and storage time

Predictor variable	Coefficient	s.e	df	*t*	*p*
(intercept)	1.860	0.087	143.641	21.280	< 0.001
Montane	− 0.305	0.089	114.187	− 3.427	< 0.001
Sarmatic	− 0.208	0.086	110.247	− 2.403	0.018
Storage time	− 0.041	0.009	60.801	− 4.349	< 0.001
Random effect (Individual ID)	0.0940, Standard deviation: 0.307				
Residuals	0.0350, Standard deviation: 0.187				

Variable coefficients are presented along with their standard errors (s.e.), degree of freedom (df), test statistics (*t*), and p-value (*p*). Reference level is the ‘boreal’ ecoregion

Statistical significance levels were set to < 0.05

## Discussion

Our results show how RTL, an indicator of chronic stress, can vary across different ecoregions. Our study provides the first assessment of telomere measurement in moose, and after controlling for sample storage duration, we show that moose from the sarmatic and montane ecoregions had shorter RTL than moose from the boreal ecoregion. These findings align with our hypothesis that moose in ecoregions encompassing higher levels of anthropogenic and environmental stress would have significantly shorter RTLs.

The characteristics of the two ecoregions with shorter RTLs vary substantially, and therefore reflect potentially different stressors that drive variation in RTL. The montane ecoregion is characterized by relatively lower land use intensity (Fig. 1), deep and extensive snow cover limiting locomotion ability, and low forage availability during winter [28]. All of which are likely responsible for elevated metabolic expenditure resulting in shorter RTL of animals in the montane ecoregion [29]. Conversely, the sarmatic ecoregion is characterized by higher moose hunting pressure from humans, competition with sympatric ungulate species and a higher land use intensity through human population density, traffic infrastructure (barriers and direct stress) and forestry activities [26–28]. The combined effect of these factors likely contributes to the shorter RTLs in this ecoregion. In addition to these anthropogenic stressors, mean annual T_c_ in the sarmatic ecoregion is substantially higher than the suggested upper critical temperature of 0 °C during winter, where moose were observed to experience increased metabolic rates and behavioural adaptation, such as altered habitat use and activity patterns [22]. When T_c_ is corrected to reflect actual ambient temperature experienced by moose in the southern ecoregion (by a conservative mean of 7.2 °C [31]), animals are on average exposed to temperatures exceeding their thermoneutral zone by approximately 7 °C during winter. This finding emphasises the concerns that moose in the southern limit of their range are heat stressed during winter [32] (Singh N. J. *personal communication*). Ultimately, chronic thermal stress [14] and trade-offs influenced by selection of suboptimal habitats [33, 34] (Singh N. J. *personal communication*) may therefore contribute towards determining RTL. Pathogen prevalence is also higher at lower latitudes with warmer climate [35, 36] and Beirne et al. [19] have demonstrated that European badgers *Meles meles* exhibit higher telomere attrition rate post infection with bovine tuberculosis. In accordance with our results, Spong et al. [21] have demonstrated that, moose hair cortisol levels—a shorter-term stress proxy than RTL—were higher in southern Sweden than in north.

RTLs of animals in the boreal ecoregion were longer compared to the other two regions. This can be attributed to a number of factors. First, the boreal region is generally more homogenous in vegetation, dominated by conifers that are interspersed with deciduous species. Commercial forestry is the main form of land use in this region, characterized by large tracts of monoculture and clear cutting being the most common method of timber harvest. Moose are known to prefer clear cuts and young pine forest < 5 m in height [37, 38]. Secondly, the proportion of migratory moose is higher in this region [28, 39], which allows the population to evade stressful periods of low food availability and deep snow, and provides food access all year round. Thirdly, the year round- availability of food through conifers being green, reduces starvation related stress. Fohringer et al. [30] identified several metabolites linked to high metabolic expenditure (e.g., several amino acids and ketone bodies) in moose in the corresponding montane area, while animals in the boreal region did not show elevated concentrations of such biomarkers that indicated starvation responses due to limiting winter diets. Moose in the montane region were observed to have a lower propensity to migrate, move shorter distances and have smaller seasonal home ranges compared to those in the boreal region [28]. This reduced migratory propensity and relatively higher and prolonged exposure to environmental stressors and a lack of abundant winter forage likely causes a higher chronic stress. The fact that all our captures were carried out during peak winter suggests that animals do not evade the environmental stressors experienced in this region at least during this period of limited browse availability [30]. Shorter RTL was also determined for roe deer [18] experiencing poor environmental conditions compared to a population in less harsh environments. Similarly, Hoelzl et al. [40] detected shorter RTL in edible dormice *Glis glis* that were not provided food ad libitum compared to individuals that were, and suggested that forage availability could be a major factor in determining telomere length in a wild species subject to highly variable resource availability.

The lack of significant results in relation to moose RTL and age in this study could be related to the fact that only animals in good body condition with the vast majority past their developmental phase, i.e., adults, were captured (Additional file 1: Figs. S1 and S2). Adult vertebrates (beyond significant additional growth) were shown to exhibit less variation in RTL than during the developmental (growing) phase [9, 13, 41]. Changes of RTL with age might, therefore, be less pronounced in adult individuals, such as those included in this study (aligning with the results of Wilbourn et al. [18] and Fairlie et al. [41] who reasoned that a selective disappearance of individuals with short telomeres increases average RTL with age in wild mammals). The onset of cellular and reproductive senescence effects in moose has been observed after the age of 10 for males [42] and 12 for females [43], however the management strategy of maintaining a moose population in prime condition, to maximise the number of individuals that can be hunted, means that few(er) individuals achieve ages at which senescence occurs. Moose management strategies in Sweden may therefore also partly explain the absence of a relationship between age and RTL. To better understand the role of animal age in telomere dynamics, individuals of all age groups would have to be examined, ideally in a longitudinal experiment [41]. Despite having observed insignificant changes of RTL with age, variation of RTL withing age groups was high and could be driven by regional effects, that may be attributed to differing degrees of environmental stress exposure and/or genetic differences. Moreover, sex was shown to not be a significant predictor for RTL in our study, which is also in line with other studies performed on free-ranging mammals (reviewed by [18, 19, 44]), but see, for instance, Watson et al. having found sex-differences in wild Soay sheep *Ovis aries* [45].

Despite the known caveats in using mammalian blood as a source material for RTL quantification, most notably due to potential immune responses causing shifts of the leukocyte profile (see [46]), we were able to rely on this sample type by streamlining lab work and careful statistical examination of potential bias-inducing variables. We were therefore able to produce comparable results in line with several other studies that relied on leukocyte DNA [e.g., 11, 18, 19, 41, 45, 47]. The strong effect of storage time highlights that telomere studies should always control for this issue if varying storage periods cannot be avoided. Reichert et al. [48] found that the storage method of blood affected RTL, indicating that storage duration will also have an effect on RTL. The effect of storage duration did not impact our study as storage time was randomly distributed throughout ecoregions and the other variables. Our study was not able to investigate whether RTL is a suitable biomarker for age (in this species and in the developmental stage tested). Future studies may benefit from the inclusion of telomerase activity estimates as suggested by several authors (e.g., [49, 50]) in order to better understand the associations of telomere length with environmental variables in the examined study system and beyond.

Due to known genetic differences between moose in southern and northern Sweden [51–53], we cannot rule out potential population effects that might contribute to differing telomere length between northern and southern ecoregions. Our finding that differences in RTL were not consistent over a latitudinal gradient is in line with Kärkkäinen et al. [16], suggesting that regional variation of telomere length may mirror local environmental conditions and/or genetic differences. By measuring heritability and including more (known) populations in their analysis, future studies should account for the effects of population pedigree [15, 17] and between-population differences on RTL [54], thereby enabling the disentanglement of potential genetic differences from environmental conditions.

## Conclusions

Animals that are highly adaptable to land use change likely face environmental constraints beyond high land use intensity that lead to an accumulation of stressors driving chronic stress and ultimately RTL. Increased encroachment via the accumulation and extension of different forms of land use and impacts of accelerated climate change at northern latitudes can limit the potential of animals to evade stressful environmental conditions via, for example, migration and will likely exacerbate metabolic demand and negative consequences on animal health. Our study emphasises that it is crucial to consider distinct biogeographic scales that encompass cumulative impacts affecting organisms holistically. Future analysis of chronic stress effects in free-ranging species should focus on the continuous resampling of cohorts of animals to understand inter and intra-individual telomere dynamics in wild animals at the life history scale.

## Methods

### Study area

The study area covers the three major ecoregions in Sweden, *i.e.* montane birch forest and grasslands (‘montane’), boreal forest (‘boreal’) and sarmatic mixed forest (‘sarmatic’) (Fig. 1; [55]). Moose were captured in all three ecoregions. Ecoregion assignment was based on the winter capture location. The ‘montane’ ecoregion is characterized by high-elevation tundra vegetation and mountain birch *Betula pubescens* belt. Duration of snow cover in the capture area within the montane ecoregion lasts approximately 210 days and mean snow depth is approximately 45 cm. Accordingly, the duration of the vegetation-growing season lasts less than 100 days in this capture area. The ‘boreal’ ecoregion occupies the largest portion of Sweden’s biomes and is dominated by coniferous trees, interspersed with patches of deciduous forest. Despite mean snow depths in capture area within the boreal being similar to the montane ecoregion, snow cover lasts less than 190 days and the growing season is extended to approximately 120 days. The ‘sarmatic’ ecoregion in southern Sweden consists of a mixed conifer-broadleaf plant association. The climate in the two capture areas within the sarmatic ecoregions is comparably mild, ranging between 90 and 200 days of snow cover, 10–15 cm snow depth and a vegetation growing period of 180–220 days. For detailed habitat characterization of moose capture areas see [28].

Forestry is the prevailing form of land use occurring throughout northern Sweden except for the montane ecoregion, where forestry is unfeasible. Generally, forestry is expected to be more intensive in the southern study area, where more commercial tree species occur and turn-over rate is higher [56]. While the landscape in the south is forest dominated, it is also highly fragmented with clear cuts, settlements and agriculture. In contrast, agriculture and settlements occur only sporadically in the boreal capture area, and are virtually absent in the montane region.

While moose and roe deer *Capreolus capreolus* occur throughout Sweden, the distribution of red deer *Cervus elaphus*, fallow deer *Dama dama* and wild boar *Sus scrofa* is limited to southern Sweden. Hunting pressure remained relatively stable for moose and roe deer in recent decades [57] but southern latitudes are experiencing higher hunting pressure due to the higher diversity of sympatric game species [26, 27]. Prevalence of disease and parasites affecting moose health was also shown to be higher in southern Sweden moose populations compared to those in the north [24, 25].

### Data collection and sampling

From 2009 to 2018, 153 samples of free-ranging adult moose were collected during winter (Jan–April) within the framework of the national moose research. Animals were immobilized from a helicopter via dart injection [58] with a CO_2_-powered rifle (Dan-Inject, Børkop, Denmark) with the drug combination of 4.5 mg etorphine (Captivon^®^ 98 Etorphine HCl 9.8 mg/ml, Wildlife Pharmaceuticals (PTY) Ltd., 38 Wilkens St., Rocky Drift, White River, South Africa) and 50 mg xylazine (Xylased^®^ 500 mg, Bioveta, a.s., Komenského 212, 68,323 Ivanovice na Hané, Česká Republica) [59–61]. During immobilization, all animals were fitted with GPS-collars including a temperature receiver (Vectronic-Aerospace, Berlin, Germany). Pregnancy status was determined by a veterinarian via rectal palpation in sarmatic and montane areas [62]. Age was estimated based on tooth wear [42, 63]. The number of calves at heel was determined visually from the helicopter. Blood samples were collected into 9 ml S-Monovette^®^ Z-Gel dry collection tubes (Sarstedt, Germany) by jugular venipuncture of the fully immobilized animals. Collection tubes were processed according to the manufacturer’s instructions and stored at − 20 °C until DNA extraction. Data on GPS positions, ancillary T_c_, sex, pregnancy status, and number of calves at heel was stored and accessed via the Wireless Remote Animal Monitoring (WRAM) database [64].

Since RTL was compared across ecoregions to evaluate chronic stress, we estimated the ambient temperature and level of human impacts experienced by moose in each of our sample areas based on their GPS tracks. Anthropogenic impacts on the landscape were measured using the global Human Modification map (gHM), which provides a cumulative measure of human modification of terrestrial lands across the globe at a 1-km resolution [29]. The mean gHM value was estimated for each individual based on one year of movement post (re-)capture. The individual movement track was standardized to eight locations per day and used to estimate the mean gHM value from the underlying raster. Moose generally show fidelity to their winter and summer ranges [28, 65] and we therefore assume that movements post-capture also reflect environmental conditions pre-capture. Similarly, mean annual T_c_ (as a proxy for ambient temperature; [31]) was based on GPS-locations post capture. We used R packages amt [66], SDLfilter [67], trajr [68], adehabitatLT and adehabitatHR [69] for GPS- and T_c_-data preparation as well as raster [70] and rgdal [71] for gHM value extraction.

### DNA extraction

Prior to DNA extraction, blood samples were thawed simultaneously at 4 °C for 4 h and the serum fraction and the gel layer were discarded. Per sample, approximately 40 mg of the coagulated blood fraction was incubated at 56 °C with 30 µl proteinase K (20 mg/ml; Qiagen, Germany) for one hour with repeated inverting and shaking of samples. A liquid state of the sample was attained by subsequent addition of 190 ml PBS pH 7.4 (2.7 mM KCl, 140 mM NaCl, 10 mM Phosphate), pipetting up and down and vortexing for 30 s. DNA extraction and purification were carried-out on a QIASymphony SP platform using the DSP DNA minikit (Qiagen, Germany) according to the manufacturer’s instructions. DNA yield and quality were quantified using a NanoDrop 2000 spectrometer (Thermo Fisher Scientific, USA; Additional file 2). Purified DNA was stored at − 20 °C for up to one month until further processing via qPCR, wherefore DNA was refrigerated at 4 °C for up to two days.

### Relative telomere length (RTL) assessment

For measuring RTL, we used the real-time PCR approach [72] adapted for moose for the first time. A 54 bp fraction of the beta-lactoglobulin (BLG) gene was used as non-variable copy number (non-VCN) gene (tested for non-variability as described by Cawthon [73], Smith et al. [74] and Turbill et al. [75]). Primer sequences for the non-VCN gene were 5′- GCA GCT GTC TTT CAG GGA GAA TG -3′ (rt_BLG F) and 5′- CCC GAC ACT TAC CAT CGA TCT TG -3′ (rt_BLG R). Telomeric primer sequences were 5′-CGG TTT GTT TGG GTT TGG GTT TGG GTT TGG GTT TGG GTT-3′ (tel 1b) and 5′-GGC TTG CCT TAC CCT TAC CCT TAC CCT TAC CCT TAC CCT-3′ (tel 2b). Telomere and non-VCN gene PCRs were carried out in 9 separate runs with 20 ng DNA per reaction, 400 nmol l^−1^ of each primer combination (Tel1b/Tel2b or rt_BLG F/ rt_BLG R) in a final volume of 20 μl containing 10 μl of GoTaq^®^ qPCR Master Mix (Promega). Samples were randomized per run based on sex, capture area, and capture year (see Additional file 2). PCR conditions for the telomere runs were 2 min at 95 °C followed by 40 cycles of 15 s at 95 °C, 20 s at 58 °C and 20 s at 72 °C. For non-VCN runs, PCR conditions were 2 min at 95 °C followed by 45 cycles of 15 s at 95 °C, 20 s at 58 °C and 20 s at 72 °C. A final melting step was included in each run with the temperature ramping from 65 to 95 °C in 1 °C steps. Each run contained a negative (non-template) control and two DNA extracts from moose livers as standard samples (to assess inter-run variability). All samples and controls were run in triplicates. Reactions were prepared using the Qiagility PCR robot (Qiagen, Germany) to minimize pipetting errors, and cycling was performed on a Rotorgene Q quantitative thermocycler (Qiagen, Germany). We used the software LinRegPCR (2012.0) [76] for analysis of non-baseline-corrected raw qPCR data, exported from the instrument. RTL was calculated using the method described by Ruijter et al. [77], modified by Hoelzl et al. [78].

The mean qPCR efficiency was calculated via the amplification plot method [76] which gives lower but more accurate estimates of efficiency than standard curve based methods [79, 80]. The estimates were 76.9% and 86.7% for the non-VCN gene and telomere reactions, respectively.

The intraclass correlation coefficient (ICC) was calculated as a measure of reliability within and between the runs, as suggested by Koo and Li [81]. ICC estimates and their 95% confident intervals for sample triplicates were calculated in R Version 3.5.2 [82]. Intra-rater ICC was calculated on all included data points based on a single-rating, absolute-agreement, 2-way mixed-effects model (ICC in library ‘irr’, [83]). Intra-assay ICC for Ct values for telomere assay was 0.85 [p < 0.0001, 95% (CI 0.82–0.88)] and for BLG 0.96 [p < 0.0001, 95% (CI 0.94–0.97)] showing a good and an excellent degree of reliability respectively. The ICC for inter-assay reliability was calculated for the standard samples based on a mean rating (k = 3), agreement, 2-way mixed-effects model. Interrater ICC for Ct values for the telomere assay was 0.94 [p < 0.0001, 95% (CI 0.54–1.0)] and for BLG 0.99 [p < 0.0001, 95% (CI 0.97–1.0)] showing an excellent degree of reliability for both. As all samples per individual were run on the same plate, inter-assay variability should have minimal effect on our longitudinal results.

The intra-assay coefficient of variation among replicates (intra-assay variation), an estimate of system precision, was further used to assess reproducibility. Mean intra-assay CV for Ct values of the non-VCN gene and telomere assay were 0.35 and 0.86%, respectively. The mean coefficient of variation among replicates (intra-assay variation) for Ct values of the non-VCN gene and telomere assay were 0.35 and 0.86%, respectively. Among runs (inter-assay variation), the mean coefficient of variation for Ct values of the non-VCN gene was 0.94%, and this was 2.76% for the telomere reaction.

### Statistics

All statistical analyses were carried out using R 3.5.2 [82]. To explain variation among individuals in RTL, linear mixed effects models and postHoc test with Tukey adjustment for multiple comparisons were used (library lme4; [84], library emmeans). The initial model contained the two-way interaction between animal age (continuous) and sex, ecoregion, as well as storage time (to control for potential effects of sample storage duration, since time of storage have been associated with change in RTL [79]) as explanatory variables. To account for potential pseudoreplication among samples from recaptured individuals, individual ID was included as a random effect. Capture location was not included in the models as they highly correlate with ecoregions that animals were captured in. Due to the limited number of samples from recaptured individuals (n = 39) and the absence of recaptures in the boreal ecoregion, intra-individual telomere dynamics were not considered in our analysis. Additionally, we ran a model on a subset of the data containing only females (n = 68), accounting for the explanatory variables mentioned above, and we also included pregnancy status and number of calves at heel as additional variables. Model selection was carried out using the R function dredge (library MuMIn; [85]) which evaluates all possible candidate models, from which the best-fit model was selected based on AICc. Coefficients, their standard errors (s.e.), degrees of freedom (df), *t* and corresponding *P*-values of the models are reported using the lmerTest package [86]. All means are given together with their standard error.

## Supplementary Information

**Additional file 1: Table S1** is the model selection table for models included in this manuscript. **Figures S1–3** are showing variables that did not pass model selection.

**Additional file 2. **DNA quality and sample distribution per qPCR run. [https://doi.org/10.5061/dryad.44j0zpcd0].

**Additional file 3. **RTL dataframe. [https://doi.org/10.5061/dryad.44j0zpcd0].

## Data Availability

The datasets generated and analysed during the current study are available in the Dryad repository, https://doi.org/10.5061/dryad.44j0zpcd0

## Abbreviations

BLG: Beta-lactoglobulin
DNA: Deoxyribonucleic acid
gHM: Global human modification
GPS: Global positioning system
HPA: Hypothalamo-pituitary-adrenal
ICC: Intraclass correlation coefficient
PCR: Polymerase chain reaction
qPCR: Quantitative polymerase chain reaction
RTL: Relative telomere length
VCN: Variable copy number
