# Supplementary material for: Large mammal telomere length variation across ecoregions
Source: BMC Ecol Evol. 2022 Aug 29;22:105. doi: 10.1186/s12862-022-02050-5 (PMC9426267; doi:10.1186/s12862-022-02050-5)
Supplement: Supplementary file 1 — Additional file 1: Table S1 is the model selection table for models included in this manuscript. Figures S1–3 are showing variables that did not pass model selection. [file 12862_2022_2050_MOESM1_ESM.docx]

**Additional file 1 for:**

Large mammal telomere length variation across ecoregions

Christian Fohringer, Franz Hoelzl, Andrew M. Allen, Claire Cayol, Göran Ericsson, Göran Spong, Steve Smith, Navinder J. Singh

**Table S1.** Model selection table for models included in this manuscript. Best model is corresponding to Table 1 in the main text. Full model, best model, and all models laying at 4 AICc difference from the lowest AICc are shown with their degree of freedom (Df).

The explanatory variables included in the full models are animal age (AgeCapt) and sex as an interactive term, ecoregion (montante, boreal, and sarmatic ecoregions where animals where captured), storage time (StTime, corresponding to sample storage duration in years). Animal ID as a random factor (1 | Object_ID) to control for potential pseudo-replication effects caused by recaptured individuals. Additional explanatory variable in the female models (where sex was excluded) were pregnancy status (Pregnancy, i.e. 0 = unknown, 1=pregnant, 2=not pregnant) and number of calves at heel (NrCalfs; 0-2).

| **Models for the prediction of Relative Telomere Length in Moose** | | **Df** | **AICc** | **Delta** |
| --- | --- | --- | --- | --- |
| Full | Ecoregion + AgeCapt * Sex + StTime | 8 | 95.2 | 3.14 |
| Best | Ecoregion + StTime | 6 | 92.1 | 0.00 |
| Other | Ecoregion + StTime + Sex | 7 | 93.8 | 1.66 |
|  | Ecoregion + AgeCapt + StorTime | 7 | 94.0 | 1.87 |
|  | Ecoregion + AgeCapt | 6 | 103.1 | 10.98 |
| Full (female) | Ecoregion + AgeCapt + Pregnancy * NrCalfs + StTime | 12 | 88.3 | 9.41 |
| Best (female) | Ecoregion + StorTime | 6 | 78.9 | 0.00 |
| Other (female) | Ecoregion + AgeCapt + StTime | 7 | 81.0 | 2.09 |
|  | Ecoregion + NrCalfs + StTime | 7 | 81.1 | 2.19 |
|  | StTime | 4 | 82.3 | 3.39 |
|  | Ecoregion + Pregnancy + StTime | 8 | 82.9 | 4.00 |

Figures S1-2 are showing variables that did not pass model selection.


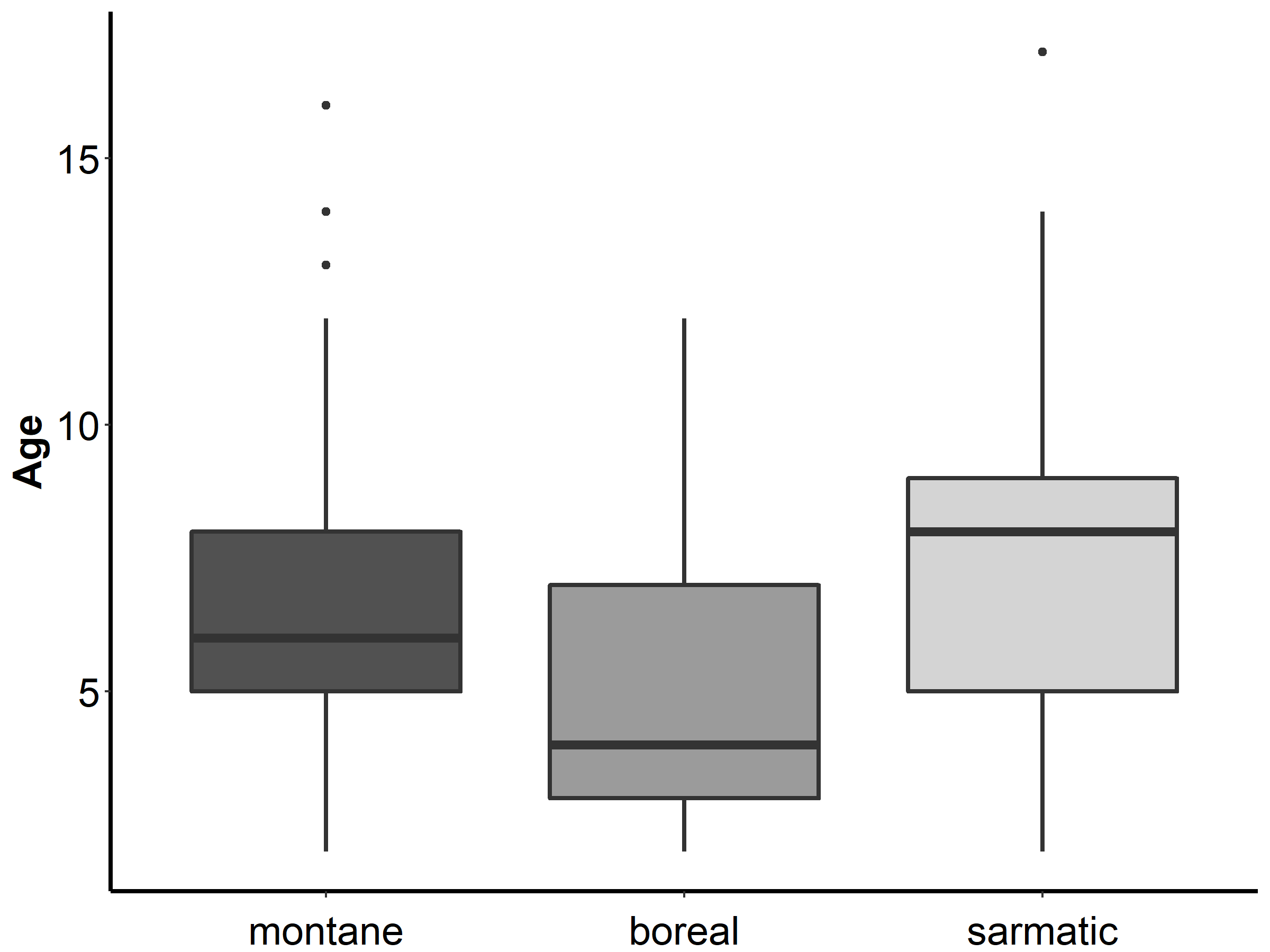


**Figure S1.** Mean age of moose captured in each ecoregion.


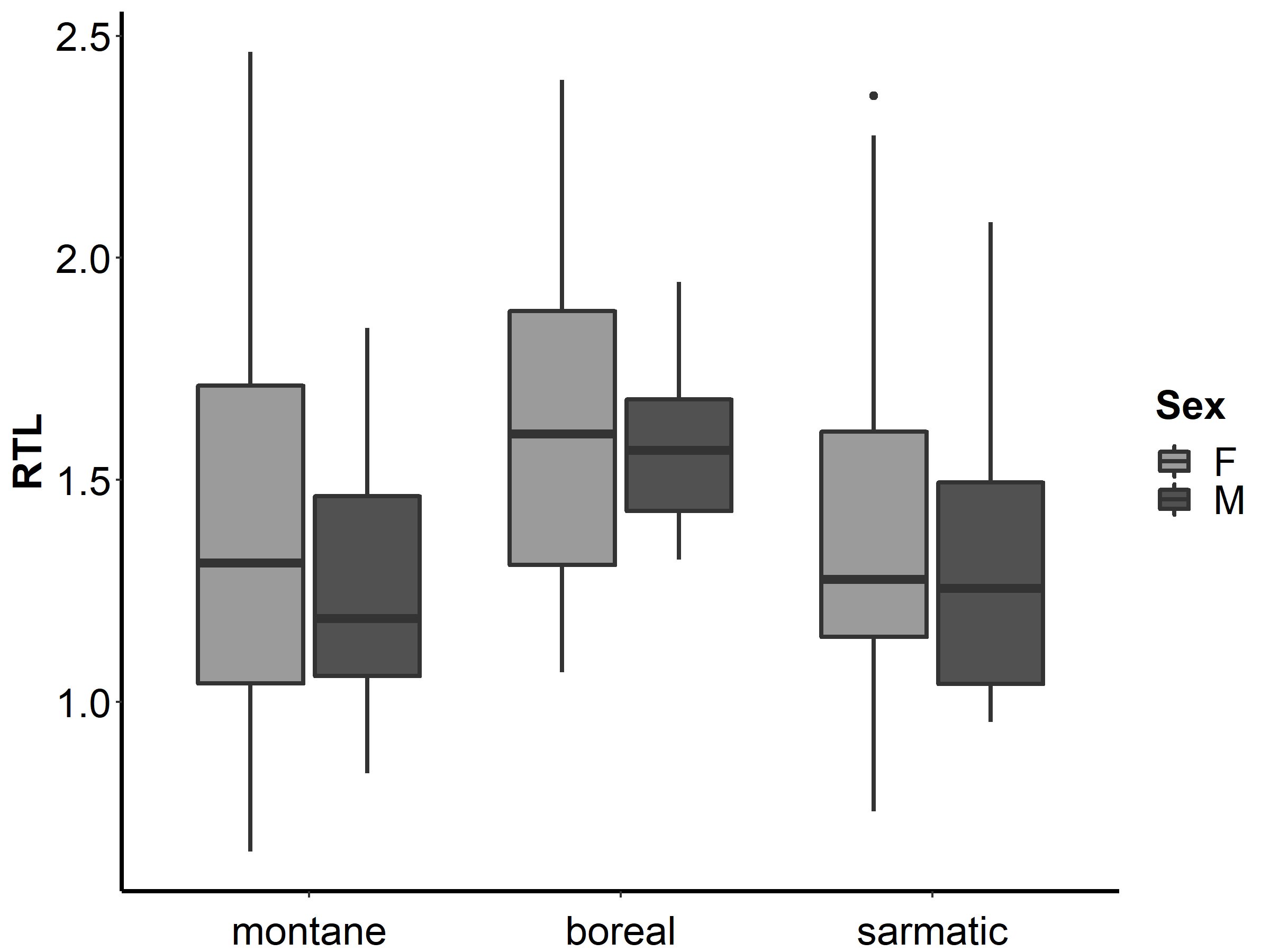


**Figure S2.** Relative telomere length (RTL) by sex for each ecoregion.

**
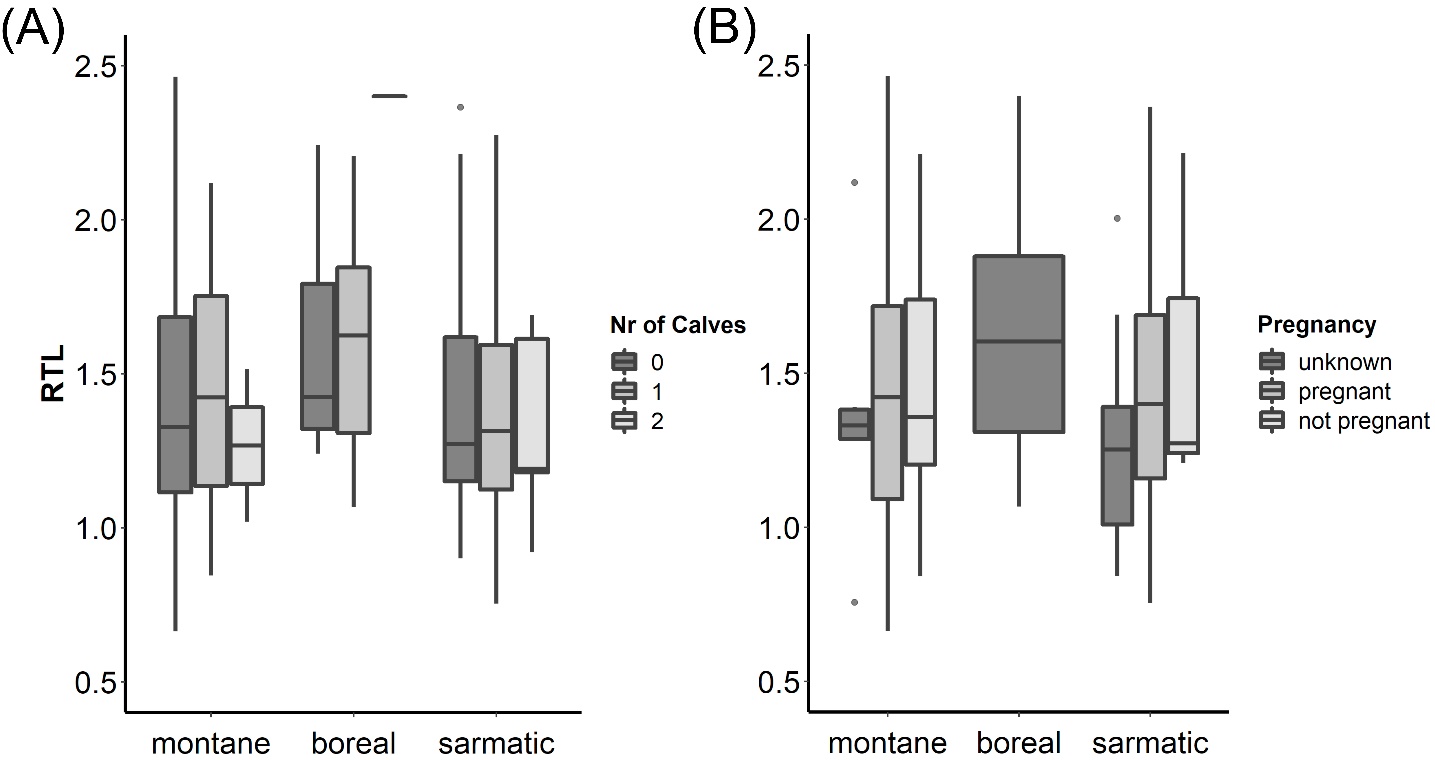
**

**Figure S3.** Relative telomere length (RTL) of females without (0), with one and with two calves at heel (A) and in relation to the pregnancy status (B) at capture, distinguished by ecoregions. Note that pregnancies were not checked during captures carried out in the boreal ecoregion.
